# GIT1 contributes to autophagy in osteoclast through disruption of the binding of Beclin1 and Bcl2 under starvation condition

**DOI:** 10.1038/s41419-018-1256-8

**Published:** 2018-12-13

**Authors:** Shu-Jie Zhao, Fan-Qi Kong, Wei Cai, Tao Xu, Zhi-Min Zhou, Zi-Bin Wang, An-Di Xu, Ya-Qing Yang, Jian Chen, Peng-Yu Tang, Qian Wang, Lin Cheng, Yong-Jun Luo, Zheng Zhou, Lin-Wei Li, Yi-Fan Huang, Xuan Zhao, Guo-Yong Yin, Ming-Xin Xue, Jin Fan

**Affiliations:** 1 Department of Orthopedics, The First Affiliated Hospital of Nanjing Medical University, Nanjing, 210000 Jiangsu China; 2 Department of Orthopedics, The Affiliated Huaian No.1 People’s Hospital of Nanjing Medical University, Huaian, 223001 Jiangsu China; 3 Analytical & Testing Center, Nanjing Medical University, Nanjing, 210000 Jiangsu China; 4 Key Laboratory of Targeted Intervention of Cardiovascular Disease, Collaborative Innovation Center for Cardiovascular Disease Translational Medicine, Nanjing Medical University, Nanjing, 210000 Jiangsu China; 5 Department of Massage, The First Affiliated Hospital of Nanjing Medical University, Nanjing, 210000 Jiangsu China

## Abstract

Approximately 10–15% of all bone fractures do not heal properly, causing patient morbidity and additional medical care expenses. Therefore, better mechanism-based fracture repair approaches are needed. In this study, a reduced number of osteoclasts (OCs) and autophagosomes/autolysosomes in OC can be observed in GPCR kinase 2-interacting protein 1 (GIT1) knockout (KO) mice on days 21 and 28 post-fracture, compared with GIT1 wild-type (GIT1 WT) mice. Furthermore, in vitro experiments revealed that GIT1 contributes to OC autophagy under starvation conditions. Mechanistically, GIT1 interacted with Beclin1 and promoted Beclin1 phosphorylation at Thr119, which induced the disruption of Beclin1 and Bcl2 binding under starvation conditions, thereby, positively regulating autophagy. Taken together, the findings suggest a previously unappreciated role of GIT1 in autophagy of OCs during fracture repair. Targeting GIT1 may be a potential therapeutic approach for bone fractures.

## Introduction

Approximately, 15 million fractures occur in the United States annually due to car accidents, sports injuries, or work accidents^1^. Furthermore, a significant proportion (10–15%) failed to heal properly, increasing socioeconomic burdens^2,3^. However, there has been slow progress in the treatment for fractures in the recent years. Fracture healing is complex and involves a variety of cells and factors, which in turn, participate in the sequential, dynamic, and intricate events of osteogenesis^4,5^. Osteoclasts (OCs) are specialized bone-resorbing cells that are abundant in the fracture callus and irreplaceable in the replacement of cartilage by woven bone^6–8^. However, the spatial and temporal regulation of OCs during bone healing is poorly understood. Hence, providing mechanism-based explanations for the healing process is expected to positively impact health.

Autophagy plays a critical role in energy and nutrient regulation and is critical in numerous pathophysiological processes^9,10^. It can be upregulated during various stress conditions, including starvation, hypoxia, and intracellular stress, where it is essential for cell survival^10,11^. In contrast, inactivation of autophagy can induce excessive degradation of proteins and eventual cell death^10,11^. Autophagosome formation and maturation are complex processes that are highly regulated by several genes^12–14^. Beclin1 plays a key role in the generation and maturation of autophagosome^15^. A complex formed by Beclin1 and class III phosphatidylinositide 3-kinase (PI3K), Vps34, can mediate autophagosome formation^15,16^. However, the activation of this complex is incompatible with the interaction between Bcl2 and Beclin1^17–19^. There is increasing evidence that autophagy plays a vital role in fracture healing^20–23^. Bone fractures impair cellular homeostasis and induce the activation of autophagy in bone cells^20–23^. However, the role of autophagy in OCs during fracture repair has not been elucidated.

G-protein-coupled receptor kinase-interacting protein 1 (GIT1) was originally identified by its binding to (G Protein-Coupled Receptor Kinase 2) GRK2^24–26^. GIT1 interacts with several signaling molecules through its functional domains and spatially regulates their localization^24–26^. Studies have reported the involvement of GIT in many fundamental cellular functions through a variety of mechanisms, including integrated signal transduction, regulation of cell polarity, cell migration regulation, and cell survival promotion^24–28^. Some studies have reported that GIT1 is also involved in bone mass regulation and fracture healing^29,30^. It is noteworthy that the OC number was comparable between GIT1 wild- type (WT) and knockout (KO) mice under normal conditions^29^. However, calluses from GIT1 KO mice showed a reduced number of OCs on day 21 following the fracture^30^. The specific mechanisms underlying GIT1-mediated regulation of the number of OCs during fracture healing are not known till date.

In the present study, reduced autophagosomes/autolysosomes in OCs and fewer OCs themselves were observed in GIT1 KO mice compared with those in the controls on days 21 and 28 during fracture healing. We also demonstrated an important role of GIT1 in promoting autophagic flux under starvation in OCs and HEK293T cells. Furthermore, the results showed that the action of GIT1 in autophagy is likely mediated through Beclin1. GIT1 physically interacted with Beclin1 and facilitated the phosphorylation of Beclin1 at the Thr119 residue, which reduced the interaction between Beclin1 and Bcl2.

## Results

### Role of GIT1 in autophagy of OCs during femoral fracture repair

**Fig. 1:**
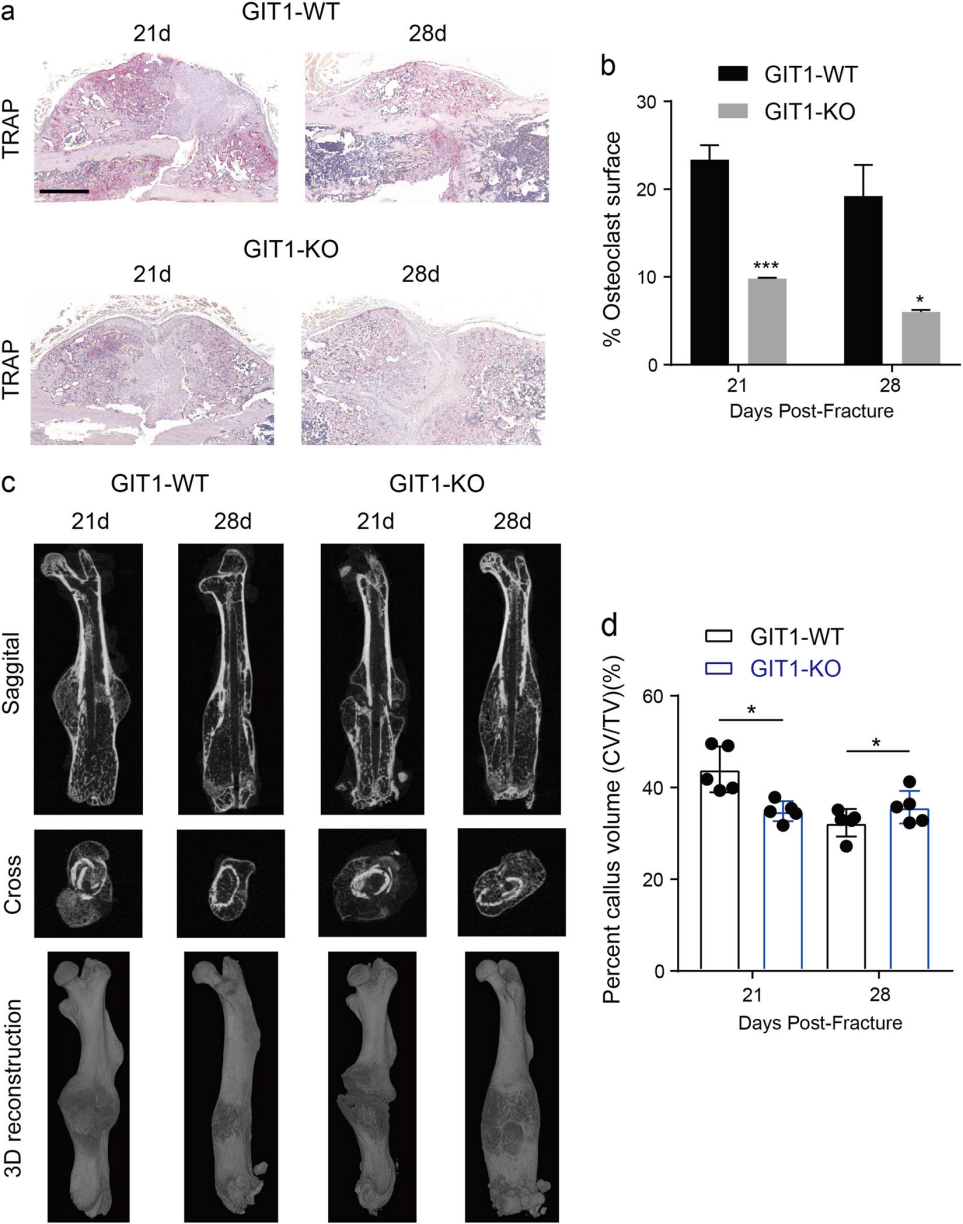
Osteoclast (OC) number and mineralized callus volume/tissue volume (CV/TV) are both affected in GIT1 KO mice. **a** Representative TRAP staining images of the femur fracture model of GIT1 WT and GIT1 KO mice on days 21 and 28 post-fracture. Scale bars = 1000 μm. **b** Statistical analysis of the osteoclast surface based on the TRAP staining in GIT1 WT and GIT1 KO mice on days 21 and 28 post-fracture (values are mean ± SD, *p < 0.05, ***p < 0.001, two-tailed Student's *t*-tests). **c** Representative 2D and 3D images from micro-CT scanning in the fracture region of GIT1 WT and GIT1 KO mice on 21 and 28 days post-fracture. **d** The statistical analysis on mineralized callus volume/tissue volume (CV/TV, %) from micro-CT scanning. Each group contained five cases (values are mean ± SD, *p < 0.05, two-tailed Student's *t*-tests)

**Fig. 2:**
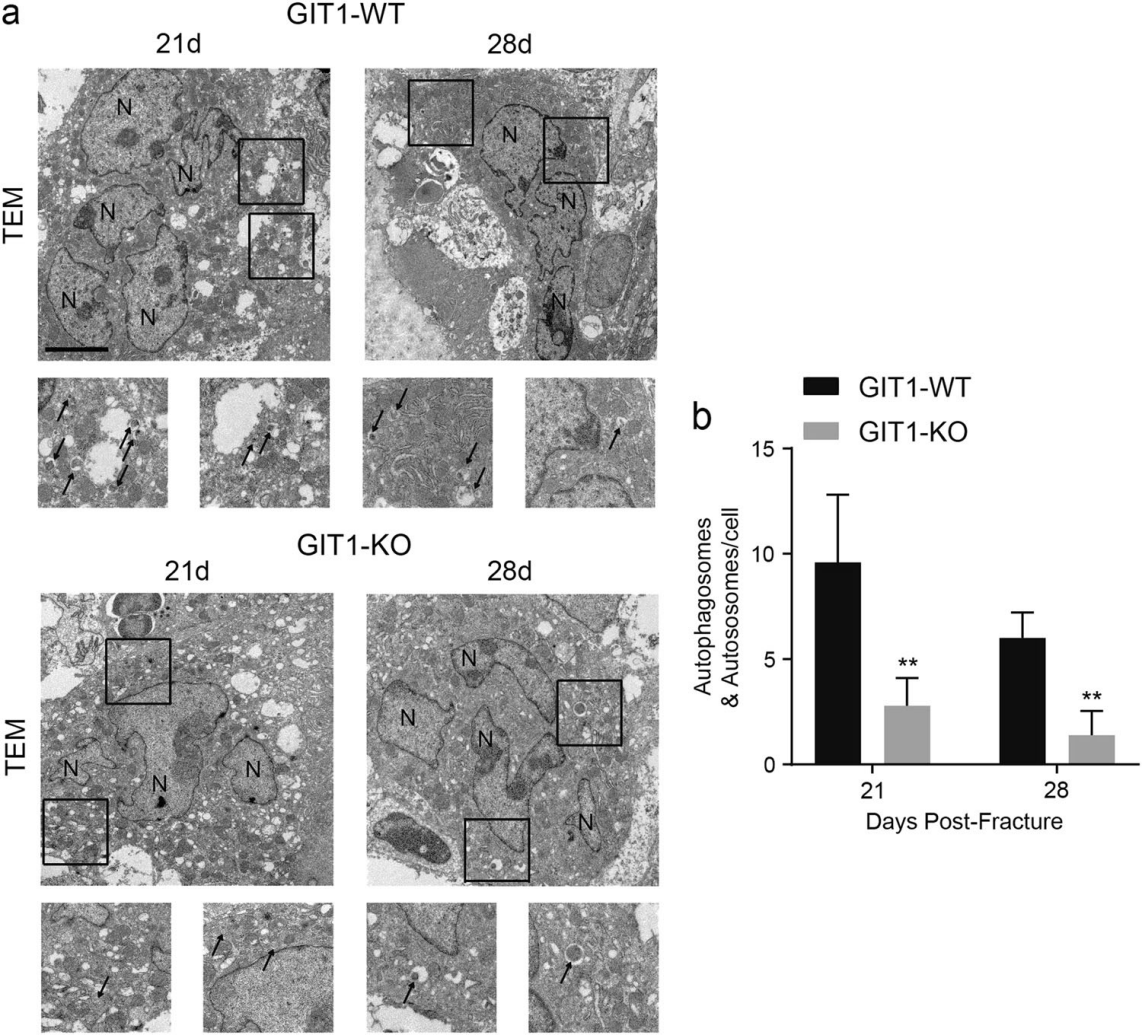
The number of autophagosomes/autolysosomes in osteoclasts is decreased in GIT1 KO mice. **a** Representative transmission electron microscopy (TEM) images of osteoclasts from the femur fracture model of GIT1 WT and GIT1 KO mice on days 21 and 28 post-fracture. Black arrows indicate autophagosomes/autolysosomes. Scale bars = 5 μm. **b** The number of autophagosomes/autolysosomes in GIT1 WT and GIT1 KO mice on days 21 and 28 during fracture repair (values are means ± SD, **p < 0.01, two-tailed Student's *t*-tests)

We used the previously generated GIT1 KO mice to explore the putative functions and mechanisms of GIT1 in OCs during fracture repair^29,30^. Supplementary Fig. 1a and b show that the number of OCs in GIT1 KO mice was comparable to that in GIT1 WT mice under basal conditions, which was consistent with the results of our previous study^29^. It is worth noting that a significantly reduced OC surface compared with that in calluses from GIT1 WT mice on days 21 and 28 post-fracture (Fig. 1a, b). Further, compared with the GIT1 WT mice, calluses from GIT1 KO mice showed a decreased mineralized callus volume/tissue volume (CV/TV) on day 21 and an increased CV/TV on day 28 post-fracture (Fig. 1c, d, and Supplementary Fig. 2). In a stress microenvironment during fracture healing, autophagy might contribute to the maintenance of cell survival^10,11^. What’s more, reduced number of OCs could cause impaired endochondral ossification and a delayed bone remodeling phase^7,8^. To further investigate the association between GIT1 and autophagy of OCs, we compared the number of autophagosomes and/or autolysosomes in GIT1 WT and GIT1 KO mice under basal or fracture healing conditions. Comparable numbers of autophagosomes/autolysosomes in OCs were observed between GIT1 WT and GIT1 KO mice under normal conditions via transmission electron microscopy (TEM) (Supplementary Fig. 1a and c). Interestingly, during fracture healing, compared with that in GIT1 WT mice, reduced numbers of autophagosomes/autolysosomes in OCs were observed in GIT1 KO mice by TEM (Fig. 2a, b). We also tested whether GIT1 affect autophagy in osteoblasts (OBs) in vivo via TEM (Supplementary Fig. 3a and b). Although, compared with GIT1 WT mice, GIT1 deletion did not reduce the numbers of autophagosomes/autolysosomes in OBs at 21 and 28 days post-fracture (Supplementary Fig. 3a and b).

Taken together, these results suggested that GIT1 may facilitate the autophagy of OCs on days 21 and 28 post-fracture.

### Knockdown of GIT1 reduced starvation-induced autophagic flux in OCs in vitro

**Fig. 3:**
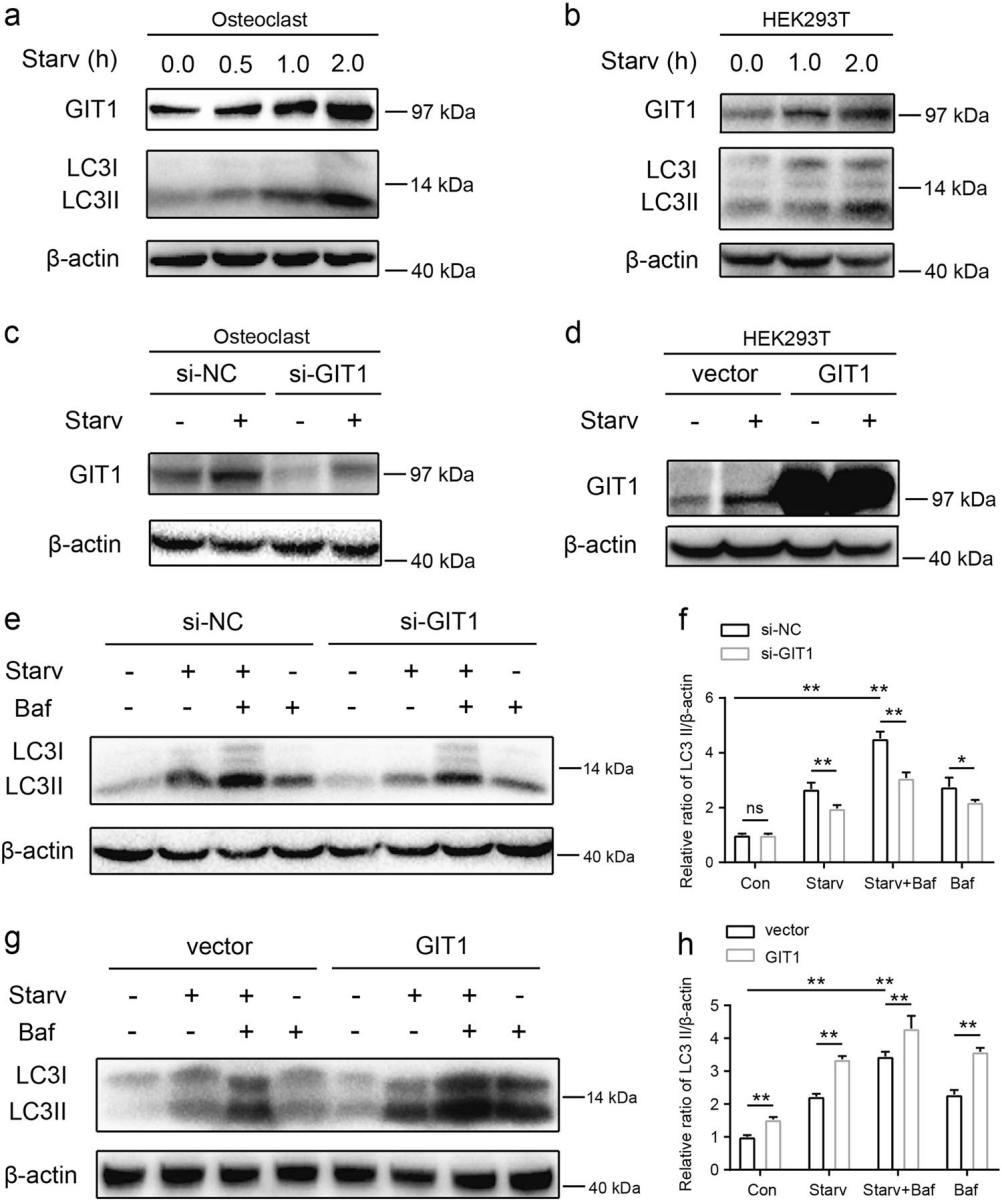
The role of GIT1 in autophagy of osteoclasts and HEK293T cells. **a** Amino-acid starvation in Earle’s balanced salt solution (EBSS) for different time durations (0, 0.5, 1.0, and 2.0 h) progressively increased the expression of GIT1 and LC3-II in osteoclasts. **b** The expression of GIT1 and LC3-II were both gradually upregulated under amino-acid starvation conditions in EBSS for different time durations (0, 1.0, and 2.0 h) in HEK293T cells. **c** The effectiveness of siRNA1 in knocking down GIT1 expression in osteoclasts under basal and starvation conditions. si-NC was used as the control. **d** The efficacy of GIT1-HA in overexpression of GIT1 in HEK293T cells under non-starvation and starvation conditions. **e**, **f** The effect of GIT1 knockdown in lowering the LC3-II level under non-starvation or starvation conditions (1 h) with or without bafilomycin A1 (Baf, 10 nM) in osteoclasts. Bafilomycin A1 was used to inhibit LC3-II degradation. Representative immunoblot images (**e**) and data summary (**f**) are shown (*p < 0.05, **p < 0.01, ns indicates no significance, Kruskal–Wallis test). **g**, **h** The effect of GIT1 overexpression in increasing the LC3-II level under basal or starvation conditions with or without bafilomycin A1 (Baf, 10 nM) in HEK293T cells. Representative images (**g**) and data summary (**h**) are shown (**p < 0.01, Kruskal–Wallis test)

To understand the interplay between GIT1 and autophagy, we explored whether GIT1 affected this process in vitro. OCs and HEK293 cells were exposed to amino-acid deprivation culture medium, Earle’s balanced salt solution (EBSS), for different time durations. As expected, amino-acid starvation caused an accumulation of microtubule-associated protein 1 light chain 3-II (LC3-II) (relative to loading controls, such as β-actin), which was correlated with the autophagosome load (Fig. 3a, b)^31^. It is noteworthy that starvation also increased the expression level of GIT1 proteins in both OCs and HEK293 cells (Fig. 3a, b).

We further determined the functions of GIT1 in the induction of autophagy in OCs. GIT1 was first knocked down in OCs by using short interfering RNA (siRNA), and the efficiency of knockdown was confirmed by quantitative PCR (qPCR) and western blotting (Supplementary Fig. 4a). The most efficient siRNA (si-3) was chosen for the following experiment. Figure 3c reveals the efficiency of GIT1 knockdown by si-3 under basal and amino-acid starvation conditions. Figures 3e, f show that the knockdown of GIT1 in OCs lowered LC3-II levels under amino-acid starvation conditions. Importantly, reduced LC3-II levels could be explained by a decrease in the autophagic induction or an increase in autolysosomal degradation^31^. We further used bafilomycin A1 (Baf A1) because it inhibited LC3-II degradation. Knockdown of GIT1 resulted in a further decrease in LC3-II levels in the presence of 10-nM Baf A1 (Fig. 3e, f). What’s more, we explored if GIT1 affect autophagic flux in OBs in vitro via western blotting. We established GIT1 knockdown cells via siRNA, and the efficiency of knockdown was tested using qPCR and western blotting (Supplementary Fig. 4b). As shown in Supplementary Fig. 4e and f, deletion of GIT1 did not significantly reduced the LC3-II levels under basal and starvation conditions with or without Baf A1. These data may suggest that GIT1 knockdown impaired autophagic flux in OCs.

**Fig. 4:**
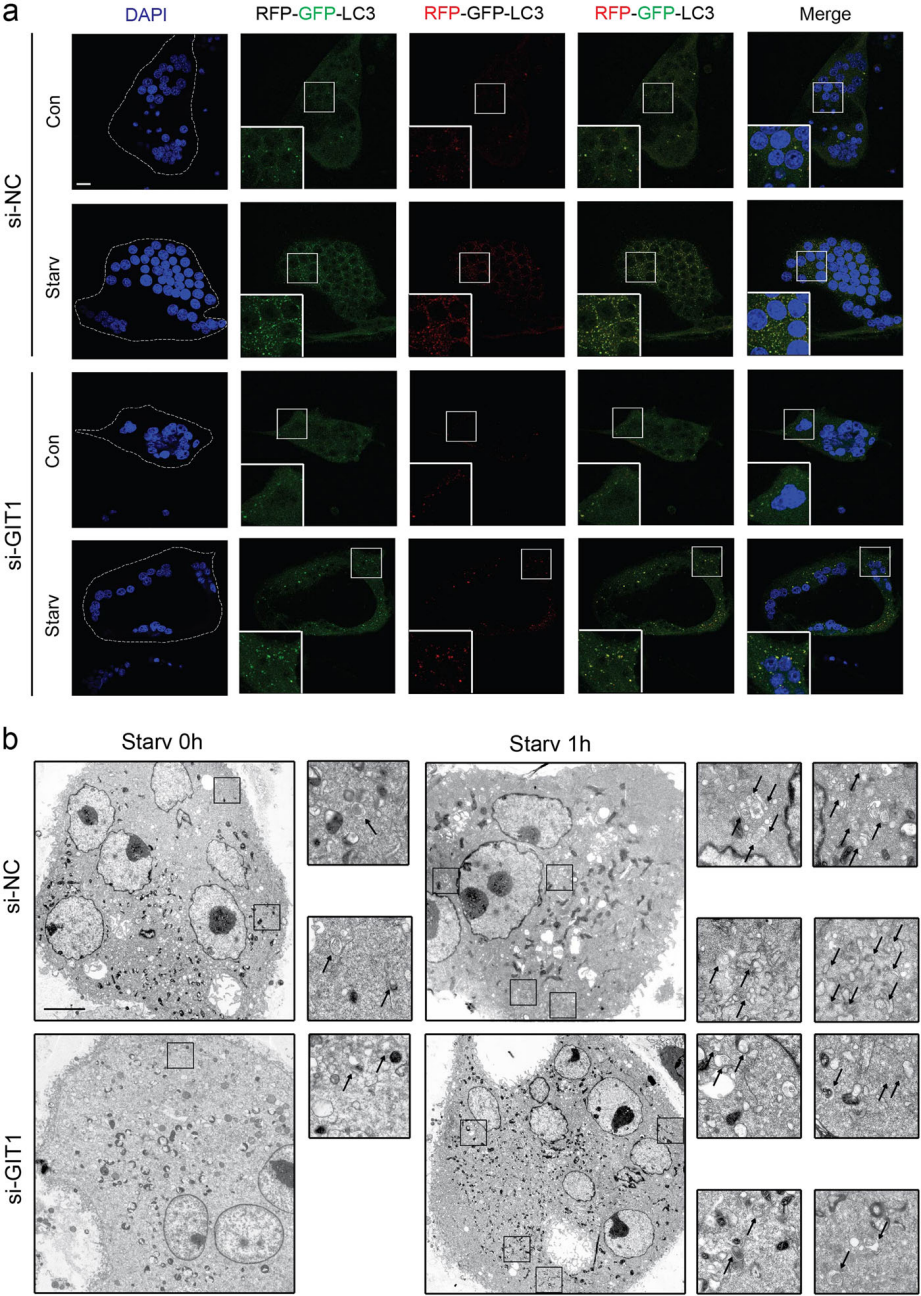
Knockdown of GIT1 inhibited the starvation-induced autophagic flux in OCs in vitro. **a** Osteoclasts were transfected with a tandem reporter monomeric red fluorescent protein (mRFP)–green fluorescent protein (GFP)–LC3. The control and GIT1 knockdown osteoclasts with the different treatments were kept in full medium, or under starvation conditions (EBSS, 1 h). Shown are LC3 fluorescent signals from representative single cells and the cell nuclei were stained with DAPI (blue). Scale bar = 20 μm. **b** The number of autophagosomes/autolysosomes of osteoclasts in GIT1 knockdown and control groups under basal and starvation conditions was analyzed via TEM. Representative images of autophagosomes/autolysosomes are shown. Black arrows indicate autophagosomes and/or autolysosomes. Scale bar = 5 μm

Subsequently, we transfected OCs with the mRFP–GFP–LC3 virus, which can effectively and conveniently monitor autophagic flux^31^. Autophagy induction results in the increase in both yellow and red puncta, inhibition of autophagy induction results in a decrease in both yellow and red puncta^31^. As expected, compared with that under basal non-starvation conditions, the number of yellow and red puncta was increased under amino-acid starvation conditions (Fig. 4a and Supplementary Fig. 4c). Importantly, the knockdown of GIT1 significantly reduced the autophagic flux under starvation, whereas it had little or no effect on autophagic flux in OCs in normal culture media (Fig. 4a and Supplementary Fig. 4c).

Moreover, the number of autophagosomes/autolysosomes was monitored by TEM. Consistent with the aforementioned results, the number of autophagosome/autolysosome increased under starvation, and the knockdown of GIT1 significantly decreased this effect (Fig. 4b and Supplementary Fig. 4d).

Collectively, these results suggested that GIT1 knockdown reduced the starvation-stimulated autophagic flux in OCs.

### Overexpression of GTI1 promoted starvation-induced autophagic flux in HEK293T cells in vitro

**Fig. 5:**
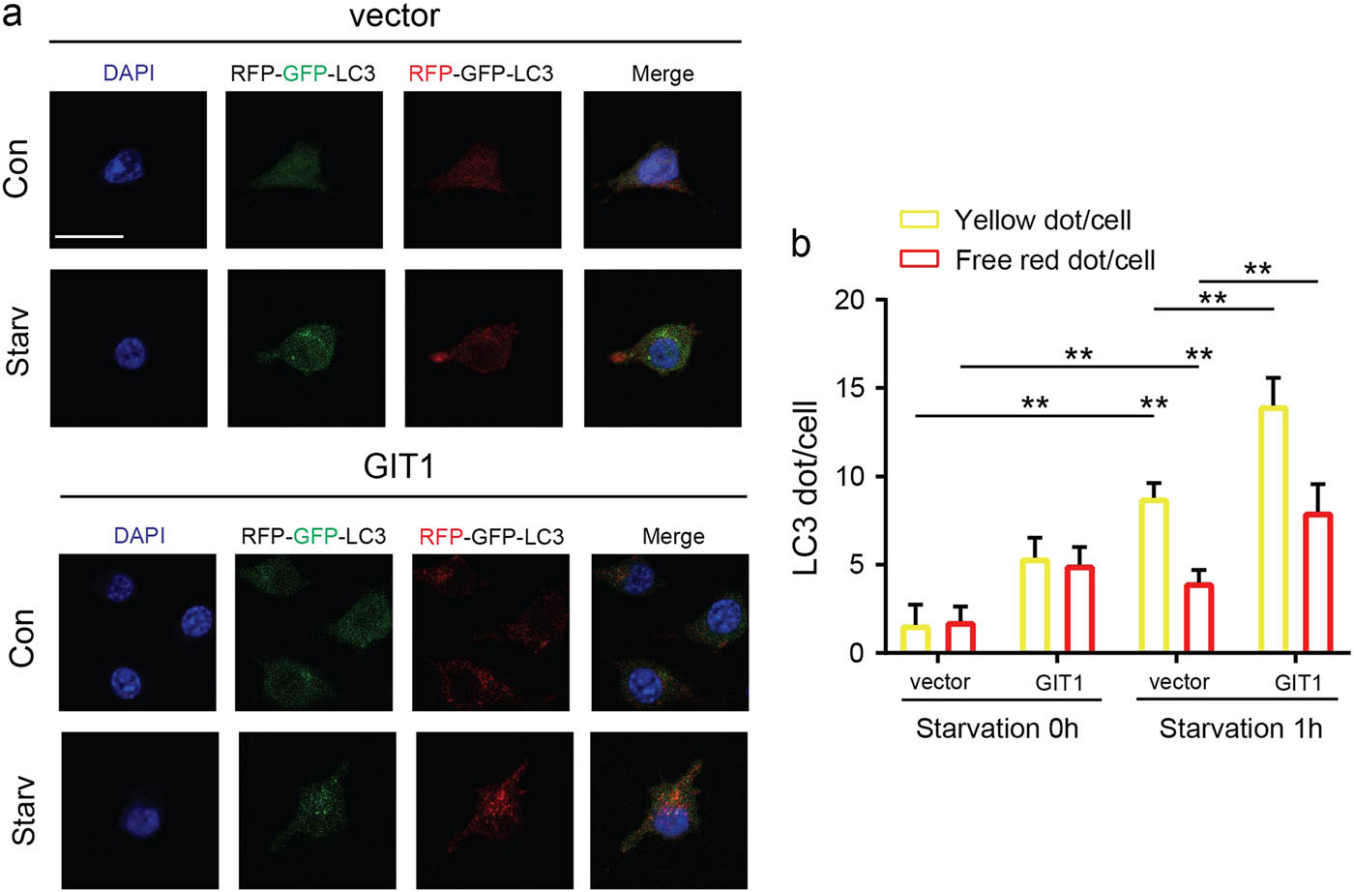
Overexpression of GIT1 promoted autophagic flux in HEK293T cells in vitro. **a, b** The control and GIT1-overexpressing HEK293T cells were transfected with mRFP–GFP–LC3. Effect of GIT1 overexpression in promoting LC3 puncta formation under non-starvation and starvation conditions. Shown are LC3 fluorescent signals from representative single cells. Representative images (**a**) and data summary (**b**) are shown (**p < 0.01, Kruskal–Wallis test). Scale bar = 20 μm

Stable GIT1-overexpressing HEK293T cells were established to further investigate the effects of GIT1 overexpression during autophagy. Western blotting was used to confirm the efficiency of GIT1 overexpression in HEK293T cells cultured in normal and starvation media (Fig. 3d). Figures 3g, h show that GIT1 overexpression increased the LC3-II levels under basal and starvation conditions with or without Baf A1. In accordance with the aforementioned results of western blotting, increased yellow puncta and red puncta can be observed in GIT1-overexpressing cells under both non-starvation and starvation conditions (Fig. 5a, b). These data indicated that overexpression of GIT1 might promote the autophagic flux in HEK293T cells.

### GIT1 facilitates Beclin1 phosphorylation at the Thr119 residue under starvation

**Fig. 6:**
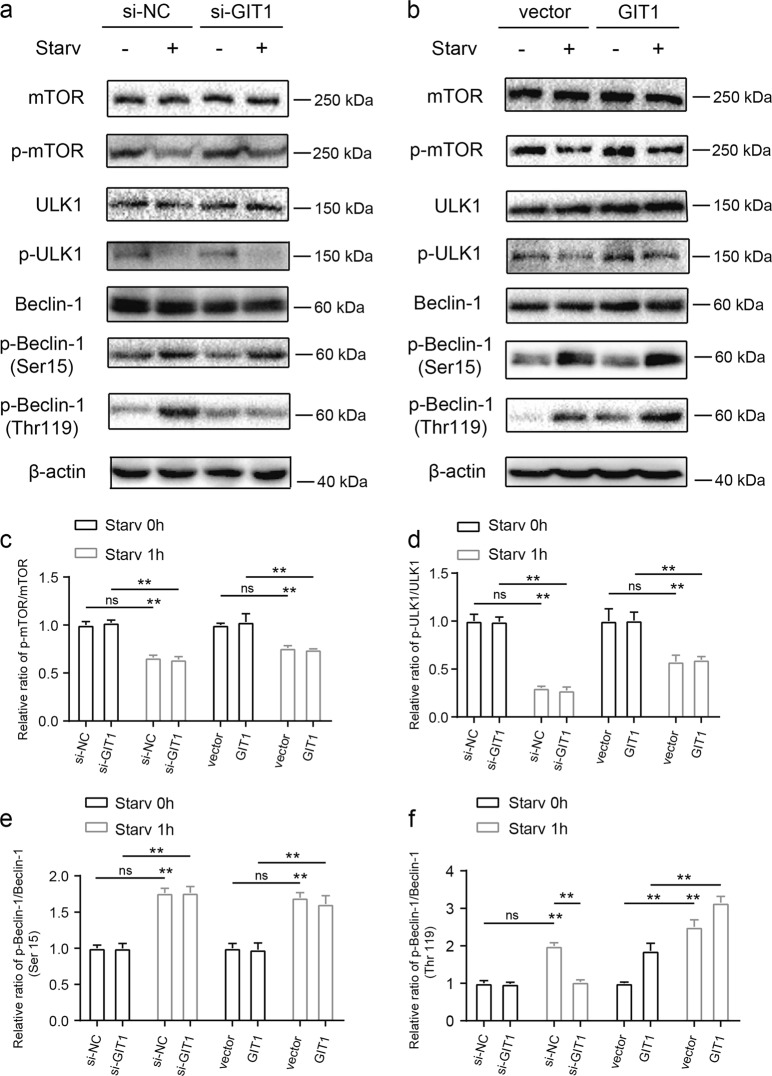
GIT1-regulated Beclin1 phosphorylation at the Thr119 residue. **a** Representative immunoblot images showing the effect of GIT1 knockdown on the expression of p-mTOR/mTOR, p-ULK1/ULK1, p-Beclin1-S15/Beclin1, and p-Beclin1-T119/Beclin1 in the OCs under basal and starvation conditions. **b** Representative immunoblot images showing the effect of GIT1 overexpression on the expression of p-mTOR/mTOR, p-ULK1/ULK1, p-Beclin1-S15/Beclin1, and p-Beclin1-T119/Beclin1 in HEK293T cells under basal and starvation conditions. **c-f** Densitometric analysis showed the relative amounts of p-mTOR/mTOR (**c**), p-ULK1/ULK1 (**d**), p-Beclin1-S15/Beclin1 (**e**), and p-Beclin1-T119/Beclin1 (**f**) in GIT1 knockdown or GIT1-overexpressing cells compared with the relative controls under basal and starvation conditions (**p < 0.01, ns indicates no significance, Kruskal–Wallis test)

To investigate the possible contributory mechanisms of GIT1 to autophagy, we first tested the possible effect of GIT1 in regulating mechanistic target of rapamycin (mTOR) activity. As expected, amino-acid starvation induced decreased phosphorylated mTOR levels, which is correlated with autophagy induction (Fig. 6a–c). As shown in Fig. 6a, c, compared with si-NC groups, the knockdown of GIT1 in OCs did not alter the level of phosphorylated mTOR in both the basal and starved conditions. A similar effect can be found in GIT1-overexpressing cells (Fig. 6b, c). We next determined whether GIT1 influences the ULK1 activity, which is a downstream phosphorylation substrate of mTOR^32^. As indicated in Fig. 6a–d, amino-acid starvation also decreased the level of phosphorylated ULK1. Like mTOR, suppression or overexpression of GIT1 failed to influence the level of phosphorylated ULK1 in both the basal non-starvation and starvation conditions (Fig. 6a–d). We tested the level of phosphorylated Beclin1 at Ser15 and Thr119 to further assess whether GIT1 was required for the regulation of Beclin1 activity. As shown in Fig. 6a–f, starvation increased phosphorylated Beclin1 at Ser15 and Thr119. Intriguingly, knockdown of GIT1 by siRNA partly attenuated the starvation-stimulated phosphorylation of Beclin1 at Thr119 but not at Ser15 (Fig. 6a–f). Furthermore, si-GIT1 had little or no effect on the phosphorylation of Beclin1 at Ser15 and Thr119 under the basal condition (Fig. 6a–f). Conversely, as shown in Fig. 6b–f, GIT1 overexpression promoted phosphorylation of Beclin1 at Thr119 but not at Ser15 under the non-starvation and starvation conditions. These data suggested that GIT1 functions to facilitate autophagy partly via regulating Beclin1 phosphorylation at Thr119, particularly under the starvation condition.

**Fig. 7:**
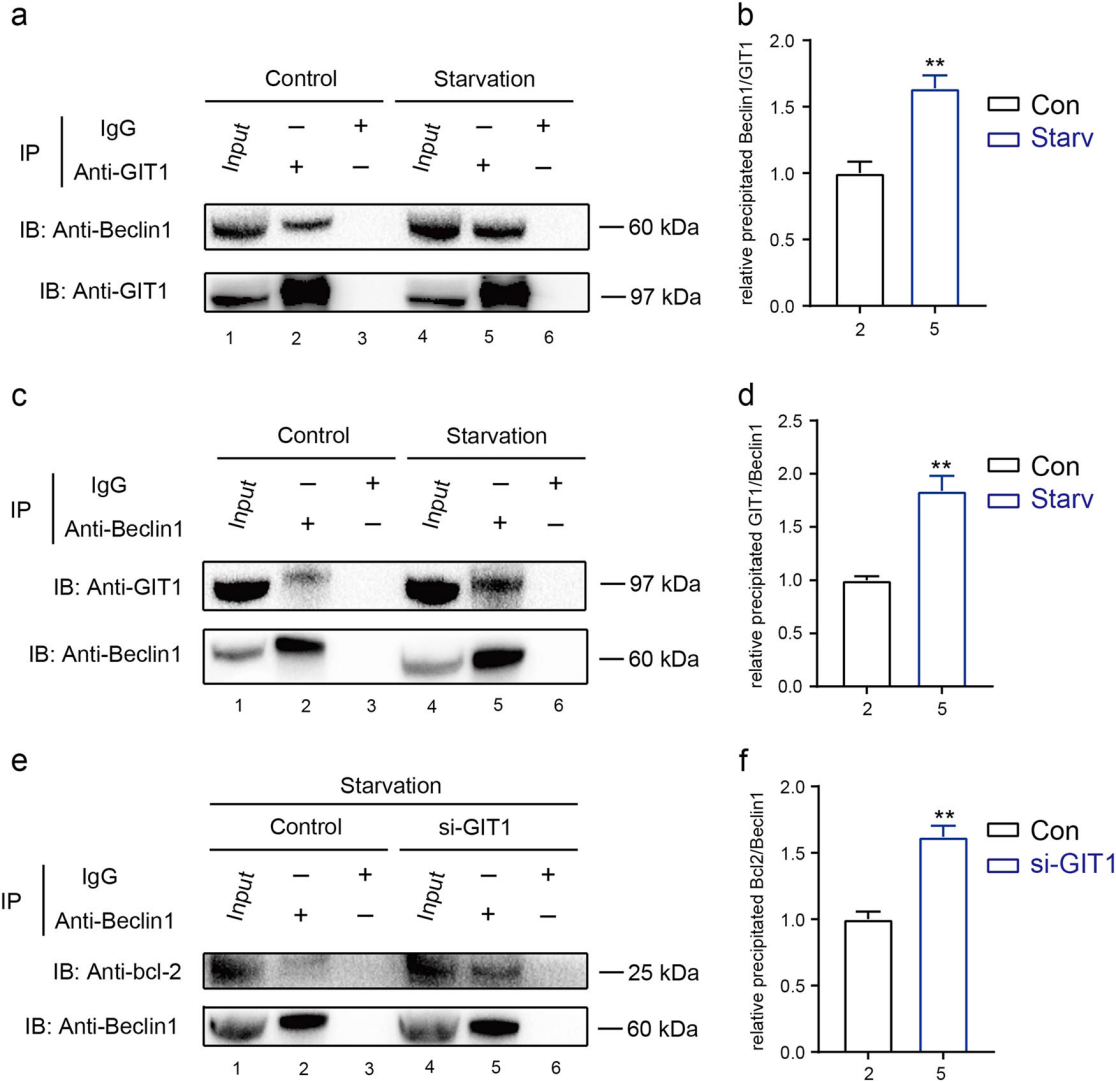
GIT1 physically interacts with Beclin1 and contributes to the disruption of Beclin1-Bcl2 binding in HEK293T cells. **a–d** Co-IP assays between GIT1 and Beclin1 under basal and starvation conditions. The pulling antibodies (anti-GIT1 antibody in **a** and anti-Beclin1 antibody in **c**) and the blotting antibodies are indicated. The cell lysates are displayed as input, and IgG is used as an internal control (values are means ± SD, **p < 0.01, two-tailed Student's *t*-tests). **e, f** Co-IP assays between Beclin1 and Bcl2 with or without GIT1 under basal and starvation conditions. The pulling antibodies (anti-Beclin1 antibody) and the blotting antibodies are indicated. The cell lysates are displayed as input, and IgG is used as an internal control (values are means ± SD, **p < 0.01, two-tailed Student's *t*-tests)

GIT1 was reported to interact with multiple signaling molecules and spatially regulated their functions^24–26^. We examined the possibility of whether GIT1 could physically interact with Beclin1 to deeply elucidate the mechanism by which GIT1 regulates Beclin1 activity. Using a co-immunoprecipitation (Co-IP) method, we found that anti-GIT1 antibody could pull-down Beclin1 proteins in both control and starved cells (Fig. 7a). Importantly, the stronger band of precipitated Beclin1 was observed in the starvation condition (Fig. 7a, b). Moreover, anti-Beclin1 antibody could reciprocally pull-down GIT1 proteins (Fig. 7c). As shown in Fig. 7c, d, this interaction was also enhanced under the starvation condition as indicated by the stronger band of precipitated GIT1. Collectively, these data suggested that GIT1 may promote Beclin1 phosphorylation at the Thr119 via an enhancer binding of GIT1 and Beclin1 in the starvation condition.

### GIT1 contributes to the disruption of Beclin1–Bcl2 binding in HEK293T cells

As a key regulator of autophagy, Beclin1 interacts with Bcl2 via its BH3 domain (amino acids 114–123), leading to downregulation of autophagy by inhibiting the formation and activation of the class III PI3K complex^17–19^. Our findings in Fig. 6a–f, indicating that GIT1 regulates Beclin1 phosphorylation at Thr119 residue, show an important post-translational modification site in Beclin1 that may promote its dissociation from the Beclin1–Bcl2 complex^15^. A Co-IP assay was performed to further confirm whether GIT1 affects interactions between Beclin1 and Bcl2 under the starvation condition. As shown in Fig. 7e, f, the interaction of Beclin1 and Bcl2 was enhanced in the absence of GIT1 as revealed by the stronger band of precipitated Bcl2. These data indicated that the interaction between Beclin1 and Bcl2 could be inhibited by GIT1 under the starvation condition in HEK293T cells.

## Discussion

Autophagy is a fundamental metabolic cellular process that enables cell survival^9^. Recently, several studies have focused on the effect of autophagy in bone physiology and pathophysiology^22,23^. Collective evidence has shown that autophagy is required to maintain the survival of the bone cell response to a stress microenvironment with insufficient nutrition at the fracture end^20–23^. Previous studies on GIT1 have suggested that it possesses diverse functions during fracture healing, including promoting chondrocyte proliferation, facilitating callus vascularity, and maintaining OC survival^30^. The idea that GIT1 contributes to OC autophagy during fracture repair was derived from the decreased numbers of OCs and autophagosomes/autolysosomes in OCs observed in GIT1 KO mice on days 21 and 28 post-fracture. However, the specific mechanisms remain to be elucidated. In this study, we elucidated a novel mechanism through which GIT1 contributes to OC autophagy under starvation conditions by phosphorylating Beclin1 at Thr119, leading to the inhibition of the interactions between Beclin1 and Bcl2.

Through its multi-domain structures, GIT1 can function as a signaling scaffold by interacting with numerous protein partners, such as Mitogen-Activated Protein Kinase Kinase (MEK), TNF Receptor Associated Factor 6 (TARF6), phospholipase C-c, p21-activated kinase-interacting exchange factor, and paxillin^24–28^. Recently, Smithson et al. reported that GIT1 can bind to mTOR, generating a new mTOR complex lacking Raptor and Rictor^33^. The interaction between GIT1 and mTOR is controlled by AKT activation and is essential for astrocyte survival^33^. Furthermore, the author found that the knockdown of GIT1 leads to a lack of changes in mTOR Ser2448 phosphorylation and LC3I/II expression under basal non-starvation conditions^33^. However, our study showed that GIT1 contributes to autophagy of OCs under starvation conditions.

Currently, the binding of Bcl2 is the best-studied negative regulatory mechanism of the autophagy function of Beclin1 in response to stress^15^. These regulatory mechanisms include (1) competitive displacement of the Beclin1 BH3 domain by other Bcl2 family proteins; the interaction could be inhibited by tBid, Bad, or BNIP3^34^, (2) competitive disruption by other Beclin1-binding proteins, such as HMGB1, UVRAG, or Atg14L may promote autophagy and cell survival during stress^15^, and (3) post-translational modifications (e.g., ubiquitination and phosphorylation) of Beclin1 or Bcl2. JUN N-Terminal Kinase 1 (JNK1) or Extracellular Signal-Regulated Kinase (ERK)-mediated phosphorylation of Bcl2 reduces the interaction between Beclin1. Death Associated Protein Kinase (DAPK) was reported to phosphorylate Beclin1 Thr119 at the BH3 domain; thus, facilitating the dissociation of Beclin1 from Bcl2-like proteins, which subsequently induces autophagy. TRAF6 and the deubiquitinating enzyme A20 regulated K63-linked ubiquitination of Beclin1 at the BH3 domain is another mechanism that triggers autophagy^15,35^. (4) Beclin1 self-interaction: Beclin1 can form homo-oligomers, which could serve as a platform for other protein–protein interactions and the displacement of Bcl2^36^. Through western blotting and Co-IP assays, our data revealed that GIT1-induced phosphorylation of Beclin1 at the Thr119 residue in its BH3 domain, promoting the dissociation of Beclin1 with Bcl2.

In our study, femoral fracture model was performed using GIT1 KO mice, whether the change in OCs in vivo was caused by the indirect effect of other cells during fracture healing after GIT1KO could not be fully determined. The exact functions and mechanisms of GIT1 in OCs in vivo require further investigations using higher specificity OCs-CKO mice. Further, we determined whether two Beclin1 phosphorylation sites were regulated by GIT1. As revealed in Fig. 6a, f, only changes in Beclin1 Thr119 phosphorylation were observed in the knockdown or overexpression of GIT1, relative to their controls. However, other phosphorylation sites possibly exist, for which antibodies are currently unavailable^37,38^. Although the interaction between GIT1 and Beclin1 was tested via Co-IP assays, the specific domain that is combined is still undetermined. In addition, the relationship between GIT1 and other Beclin1-interacted proteins in autophagy is unknown. Advances in this field will help to further decipher the complex roles of GIT1 in the progression of autophagy.

In conclusion, we demonstrated that the number of OCs and autophagosomes/autolysosomes were reduced in GIT1 KO mice compared with those in GIT1 WT mice on days 21 and 28 post-fracture. Furthermore, GIT1 is required for autophagy in OCs and HEK293T cells under starvation conditions. GIT1-induced phosphorylation of Beclin1 at Thr119 in its BH3 domain induces the disruption of the molecular association between Beclin1 and Bcl2 under starvation conditions; thereby, positively regulating autophagy.

## Materials and methods

### Cell culture and reagents

HEK293T, RAW264.7 and hFOB1.19 cells were all purchased from the Cell Bank of the Chinese Academy of Sciences (Shanghai, China). Each cell line passed the test of DNA profiling (short tandem repeat (STR) profiling method). The mycoplasma contamination testing was performed using Mycoplasma Genus polymerase chain reactor (PCR). For OC differentiation analyses in vitro, RAW264.7 cells were stimulated with 50 ng/mL RANKL (462-TEC, R&D, MN, USA) for 4 days. For in vitro cell starvation experiments, cells were counted before seeding in the plate to ensure the same cell number between the different cell groups. Starvation was performed in EBSS (B610KJ, BasalMedia, Shanghai, China). If needed, Baf A1 (10 nM) (HY-100558, MCE, NJ, USA) was added in the cell medium for 1 h before EBSS treatment. The antibodies in this study included anti-β-actin (AB0011, Abways, Shanghai, China), anti-GIT1 (NBP2–22423, Novus, CO, USA), anti-mTOR (2972, CST, MA, USA), anti-p-mTOR (5536, CST), anti-ULK1 (8054, CST), anti-p-ULK1 (6888, CST), anti-LC3-II (NB100–2220, Novus), anti-Beclin1 (3738, CST), anti-p-Beclin1 (Ser15) (84966, CST), anti-p-Beclin1 (Thr119) (AP3765a, Abgent, CA, USA), anti-Bcl2 (3498, CST), anti-rabbit IgG (2729, CST), and anti-mouse IgG (ab188776, Abcam, Cambridge, UK). Secondary antibodies were anti-mouse IgG (H + L) (115–035–003, Jackson ImmunoResearch, PA, USA), anti-rabbit IgG (H + L) (111–035–003, Jackson ImmunoResearch), anti-rabbit IgG light chain (ab99697, Abcam), anti-rabbit IgG heavy chain (ab99702, Abcam), and anti-mouse IgG light chain (A25012, Abbkine, CA, USA).

### RNA isolation and qPCR

In accordance with the manufacturer’s instructions, TRIzol reagent (Takara, Dalian, China) was used to extract the total RNA, and the PrimeScript reverse transcription-polymerase chain reaction (RT-PCR) kit (Takara) was used to perform the RT. The primer sequences are listed in Supplementary Table 1. The qPCR was performed using a 7500 real-time PCR system (Applied Biosystems, Inc., USA), and quantification of all gene transcripts was performed with the SYBR® Premix Ex Taq™ kit (Takara Bio, Ōtsu, Japan). The settings of the thermal cycler program have been previously described^39^. Glyceraldehyde-3-phosphate dehydrogenase (GAPDH) served as the internal control.

### siRNA transfection

Cells were cultured at 60% confluence and transfected with GIT1-specific small interfering (si) RNA (GenePharma, Shanghai, China) using Lipofectamine® RNAiMAX (Thermo Fisher Scientific, Waltham, MA, USA). A non-targeted siRNA (si-NC) was used as the control. The transfection steps were performed based on the manufacturer’s protocols. The sequences of these siRNAs are listed in Supplementary Table 1.

### Plasmid construction and transfection

The plasmid containing GIT1-HA and a negative control plasmid were obtained from FulenGen Ltd., Co. (Guangzhou, China). GIT1-HA and mock vector were both packaged into the virus, and titers were determined. The cells were infected with 1 × 10^8^ lentivirus-transducing units and 5 μg/mL polybrene (Sigma-Aldrich, Shanghai, China). After 72 h, the infected cells were screened in the presence of 2 μg/mL puromycin. The qPCR and western blotting (WB) verified the overexpression efficacy of GIT1.

### LC3 puncta quantification

The mRFP–GFP–LC3 virus was purchased from Hanbio Biotechnology Co., Ltd. (Shanghai, China), and titers were determined (1 × 10^8^). The cells were first transfected with the mRFP–GFP–LC3 virus. After 48 h, the cells were starved in EBSS for 0 or 1 h. The nuclei were counterstained with 4',6-Diamidino-2-Phenylindole (DAPI) (Sigma-Aldrich). The images were taken using a Confocal Imaging System (Zeiss LSM710, ZEISS, German). Formation of autophagosomes causes an increase in the number of GFP-positive/mRFP-positive (yellow) puncta, and the puncta become GFP-negative/mRFP-positive (red) upon fusion with lysosomes.

### Femoral fracture model and X-ray imaging

GIT1 KO mice (C57BL/6 background) were generated as previously described^29,30^. WT littermates (GIT1 WT) were used as controls. All mice were reared and handled in compliance with the Animal Committee at the First Affiliated Hospital of Nanjing Medical University. The femoral fracture model was also performed as described previously^30^. In brief, the mice (10–12 weeks) were first anesthetized. Subsequently, a 10-mm incision was made, and a Kirschner’s wire was inserted through the patellar tendon into the femoral marrow cavity. Bone forceps was used to create a mid-diaphyseal fracture. Furthermore, the wound was closed by suture, and the mice were injected with buprenorphine daily postoperatively for 3 days to control pain. An X-ray system (MX-20, Faxitron, USA) was used to observe the callus 21 and 28 days after the fracture. Subsequently, the femurs were harvested, fixed with 4% paraformaldehyde for 24 h, decalcified in 10% ethylenediaminetetraacetic acid (EDTA) for 21–28 days, and embedded with paraffin.

### Microcomputed tomography imaging (micro-CT) imaging

After removing the Kirschner’s wire, femora were fixed in 4% paraformaldehyde for 24 h and then scanned with micro-CT system (SkyScan 1176, Bruker, Germany) at a resolution of 18 µm and with the settings 50 kV and 200 µA. The three-dimensional structures were constructed and the bone morphometric parameters (mineralized CV/TV) were analyzed with CT-Analyser (CTAn, Bruker, Germany).

### Tartrate-resistant acid phosphatase staining

Tartrate-resistant acid phosphatase (TRAP) staining for callus was performed, as previously described^29,30^. In brief, decalcified sections were first deparaffinized and preincubated. Subsequently, the slides were incubated for 45–60 min at 37 °C with TRAP staining solution, based on the manufacturer’s protocol (387A-1KT, Sigma-Aldrich, MO, USA). The slides were also counterstained with hematoxylin solution. For quantification, the OC surface was calculated using an image processing program (ImageJ software, National Institutes of Health, Bethesda, MD, USA).

### TEM

The cells were collected after centrifugation (1000 *g*, 5 min). Subsequently, the cells were washed twice with cold phosphate-buffered saline (PBS) and fixed with 2.5% glutaraldehyde overnight at 4 °C. Notably, callus tissues must be decalcified in 10% EDTA for 28 days at room temperature (20 °C). A secondary fixation was performed in a solution of 1% osmium tetroxide for 1 h. Subsequently, the samples were stained in 70% ethanol-saturated uranyl acetate, dehydrated with ethanol–acetone (series of dilutions), and embedded using epoxy resin 812. The samples were sectioned at 70 nm, stained with uranyl acetate, followed by lead citrate for 5 min, and photographed using a TEM (Tecnai G2 Spirit Bio TWIN, FEI, USA).

### Western blotting and Co-IP

Western blotting was performed as previously described^39^. Briefly, total cellular protein was extracted, and equal amounts of proteins were subjected to sodium dodecyl sulfate–polyacrylamide gel electrophoresis and transferred onto a polyvinylidene fluoride membrane. After blocking with 5% skimmed milk (detection of non-phosphorylated protein) or 5% Bovine Serum Albumin (BSA) (detection of phosphorylated protein), the membranes were incubated with the following specific antibodies: anti-β-actin (1:2000), anti-GIT1 (1:1000), anti-mTOR (1:1000), anti-p-mTOR (1:1000), anti-ULK1 (1:1000), anti-p-ULK1 (1:1000), anti-LC3-II (1:1000), anti-Beclin1 (1:1000), anti-p-Beclin1 (Ser15) (1:1000), and anti-p-Beclin1 (Thr119) (1:1000) overnight at 4 °C. Immunodetection was accomplished with species-specific secondary antibodies (1:10000), followed by western ECL (electro-chemi-luminescence) substrate (Share-bio, Shanghai, China).

For the Co-IP assay, whole-cell lysates were prepared as mentioned above. Target proteins (Beclin1, GIT1, and Bcl2) were immunoprecipitated by incubating 800 μg of the extracted proteins with anti-GIT1 or anti-Beclin1 antibody, respectively, at 4 °C with rotation overnight. Protein A/G Sepharose (Share-bio, Shanghai, China) was then added and incubated for 2 h on a spinning wheel at 4 °C. Subsequently, the beads–antibody complex and protein lysate were suspended. The beads were collected by centrifugation at 3000 *g*, followed by three washes with lysis buffer. Following this, the immunoprecipitates were analyzed using western blot. Secondary antibodies such as anti-rabbit IgG light chain (1:10000), anti-rabbit IgG heavy chain (1:2000), and anti-mouse IgG light chain (1:5000) were used.

### Statistical analyses

Data are shown as mean ± standard deviation (SD) for at least three independent experiments. GraphPad Prism 7 (GraphPad Software, La Jolla, CA, USA) was used to determine statistical analyses. Comparisons between groups were performed using two-tailed Student's *t*-tests. Furthermore, Kruskal–Wallis tests were performed for comparison of more than two groups. Values of *p* < 0.05 were considered statistically significant.

## Supplementary information

Supplementary Figure Legend

Supplementary Figure 1

Supplementary Figure 2

Supplementary Figure 3

Supplementary Figure 4

Supplementary Table 1
