# Supplementary material for: GIT1 contributes to autophagy in osteoclast through disruption of the binding of Beclin1 and Bcl2 under starvation condition
Source: Cell Death Dis. 2018 Dec 13;9(12):1195. doi: 10.1038/s41419-018-1256-8 (PMC6294144; doi:10.1038/s41419-018-1256-8)
Supplement: Supplementary file 1 — Supplementary Figure Legend [file 41419_2018_1256_MOESM1_ESM.doc]

**Supplementary Fig 1**

(a) Representative TRAP staining, and transmission electron microscopy (TEM) images of the femur sections of GIT1 WT and KO mice. Black arrows indicate autophagosomes/autolysosomes in TEM images. Scale bars = 200 μm for TRAP staining; Scale bars = 2 μm for TEM.

(b) Statistical analysis of ostoeclast/mm^2^ was quantified using light microscopy in GIT1 WT and GIT1 KO mice. Values are means ± SD, ns indicates no significance.

(c) The number of autophagosomes/autolysosomes in GIT1 WT and GIT1 KO groups via TEM. Values are means ± SD, ns indicates no significance.

**Supplementary Fig 2**

(a) Representative radiograph images of the femur fracture model of GIT1 WT and GIT1 KO mice on days 21 and 28 post-fracture.

**Supplementary Fig 3**

(a) Representative TEM images of osteoblasts from the femur callus of GIT1 WT and KO mice. Black arrows indicate autophagosomes/autolysosomes in TEM images. Scale bars = 2 μm.

(b) The number of autophagosomes/autolysosomes in osteoblasts of GIT1 WT and GIT1 KO mice on days 21 and 28 during fracture repair (Values are means ± SD, ns indicates no significance, two-tailed Student t-tests).

**Supplementary Fig 4**

(a and b) Knockdown efficacy of GIT1 in osteoclasts (a) and osteoblasts (b) was determined by real-time qPCR (n = 3) and western blotting. Values are means ± SD, **p < 0.01, two-tailed Student t-tests.

(c) The control and GIT1 knockdown osteoclasts were transfected with mRFP-GFP-LC3. Statistical analysis of LC3 puncta in control and GIT1 knockdown groups under basal and starvation conditions (**p <0.01, ns indicates no significance, Kruskal-Wallis test).

(d) The number of autophagosomes/autolysosomes of osteoclasts in GIT1 knockdown and control groups under basal and starvation conditions was analyzed via TEM. Values are means ± SD, *p < 0.05, **p < 0.01, two-tailed Student t-tests.

(e and f) The effect of GIT1 knockdown in LC3-II accumulation under basal or starvation conditions (1 h) with or without bafilomycin A1 (Baf, 10 nM) in hFOB1.19 cells. Representative immunoblot images (e) and data summary (f) are shown (**p <0.01, ns indicates no significance, Kruskal-Wallis test).
