# Supplementary figures and images for: GIT1 contributes to autophagy in osteoclast through disruption of the binding of Beclin1 and Bcl2 under starvation condition
Source: Cell Death Dis. 2018 Dec 13;9(12):1195. doi: 10.1038/s41419-018-1256-8 (PMC6294144; doi:10.1038/s41419-018-1256-8)

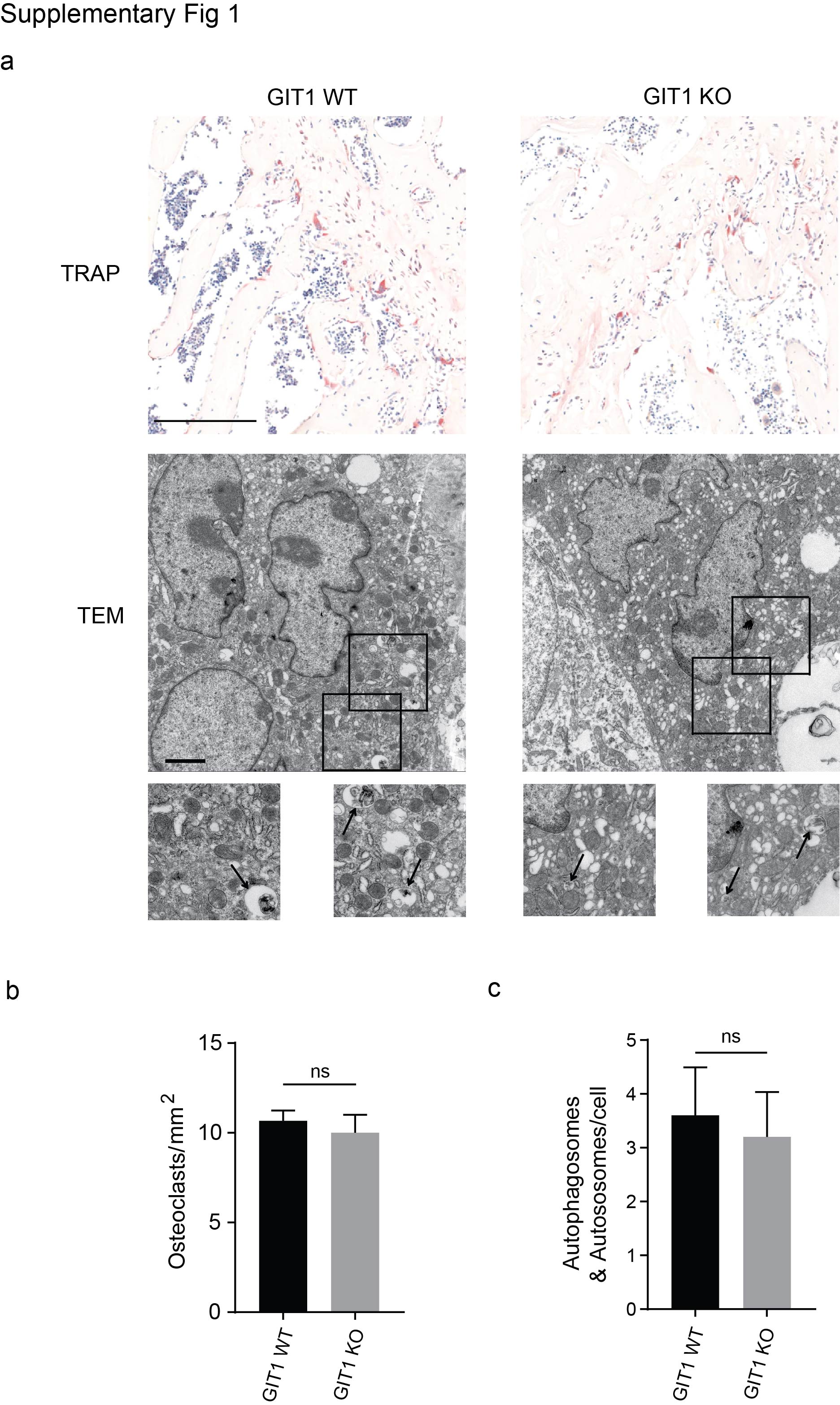

Supplement: Supplementary file 2 — Supplementary Figure 1 [file 41419_2018_1256_MOESM2_ESM.jpg]

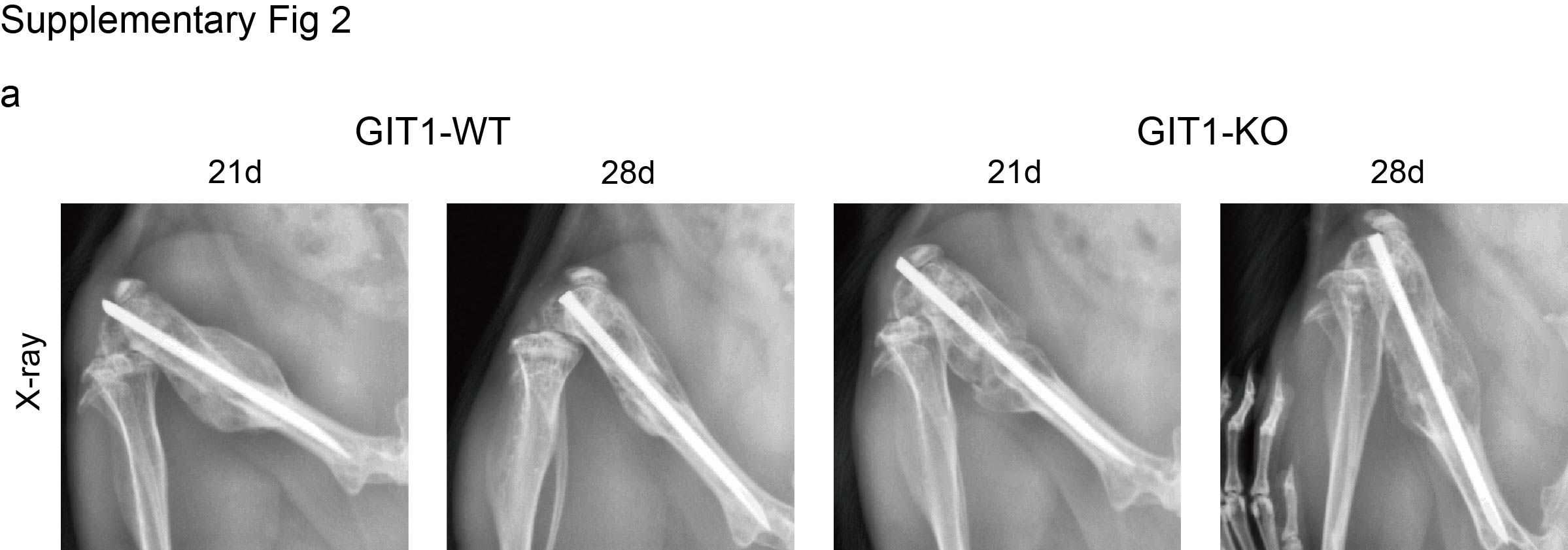

Supplement: Supplementary file 3 — Supplementary Figure 2 [file 41419_2018_1256_MOESM3_ESM.jpg]

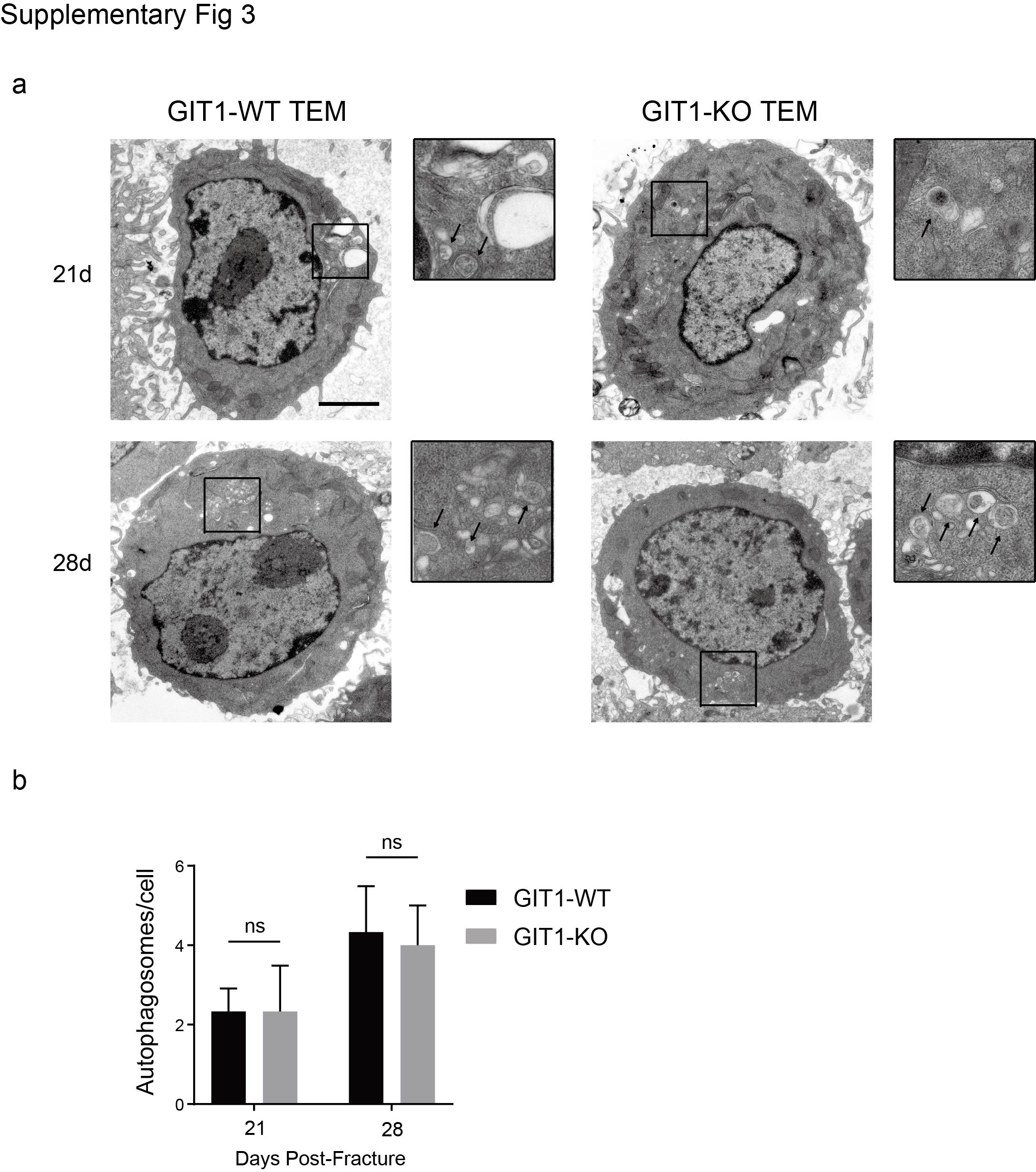

Supplement: Supplementary file 4 — Supplementary Figure 3 [file 41419_2018_1256_MOESM4_ESM.jpg]

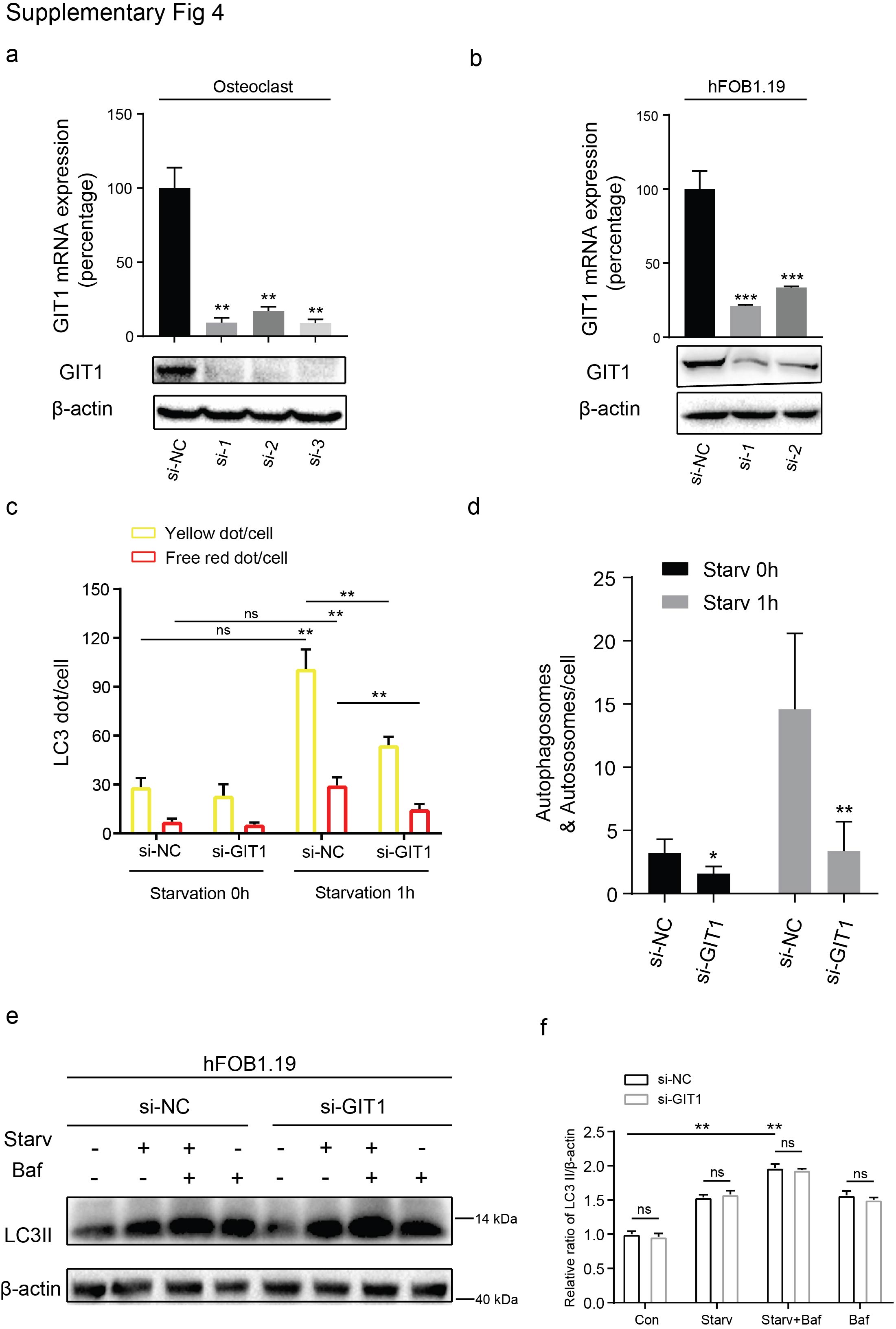

Supplement: Supplementary file 5 — Supplementary Figure 4 [file 41419_2018_1256_MOESM5_ESM.jpg]
